# Association between socioeconomic status and post-stroke depression in middle-aged and older adults: results from the China health and retirement longitudinal study

**DOI:** 10.1186/s12889-024-18503-z

**Published:** 2024-04-11

**Authors:** Qianru Cai, Mengyi Qian, Meiling Chen

**Affiliations:** 1 The Second School of Clinical Medicine, Zhejiang Chinese Medical University, Hangzhou, China; 2 School of Humanities and Management, Zhejiang Chinese Medical University, Hangzhou, China; 3 Institute of Zhejiang Chinese Medical Culture, Zhejiang Chinese Medical University, Hangzhou, China

**Keywords:** Stroke, Depression, PSD, Socioeconomic status, Middle-aged and older adults

## Abstract

**Introduction:**

Post-stroke depression (PSD) is a common neuropsychiatric complication that affects approximately one-third of stroke patients. The treatment and prognosis of this disease are poor. Socioeconomic status (SES) is closely related to health outcomes; however, only a few previous studies have focused on the association between SES and PSD. Given the substantial population of stroke patients in China, it is crucial to examine the potential risk factors associated with PSD. Conducting studies on this population and investigating the influence of economic conditions can provide valuable guiding theoretical insights into PSD prevention and management.

**Methods:**

We used data from the 2018 China Health and Retirement Longitudinal Study and selected appropriate samples for analysis. Depression was estimated using the Center of Epidemiologic Studies Depression Scale-10, a validated tool for assessing depression in the general population. Multiple logistic regression analysis was employed to assess the association between SES and PSD and to evaluate any urban–rural differences.

**Results:**

Of the 749 respondents, 370 (49.4%) had depression. Stroke patients with a middle school education demonstrated a greater risk of developing depression than those with a primary school education or below after adjusting for all control variables (odds ratio (OR) = 1.60, 95% confidence interval (CI): 1.03–2.51, *P* = 0.036). However, stroke patients with a high school education or above had a lower risk of developing depression than those with a primary school education or below (OR = 0.50, 95% CI: 0.28–0.88, *P* = 0.016). In rural areas, stroke patients with a high school or above education level had lower rates of depression than those with a primary school education or below (OR = 0.44, 95% CI: 0.21–0.91, *P* = 0.027). This difference was not significant in urban areas.

**Conclusions:**

SES significantly influences the occurrence of PSD, which is reflected by education attainment and annual household expenditures. Education attainment was an independent influence on PSD, with a more pronounced effect in rural versus urban areas. We hope to reduce the prevalence of PSD and enhance the comprehensive management of this disease by modifying the influencing factors. Sex, self-reported health status, activities of daily living, night-time sleep duration, and life satisfaction also influenced the occurrence of PSD.

**Supplementary Information:**

The online version contains supplementary material available at 10.1186/s12889-024-18503-z.

## Introduction

 Stroke, a major global health concern, is the second leading cause of death and the third leading cause of disability and death worldwide [1]. Between 1990 and 2019, stroke-related deaths increased by 43%, and disability-adjusted life years due to stroke increased by 32% [1]. Many stroke patients experience neurological impairments that affect their daily functioning and work capacity and increase their families’ economic and caregiving burdens [2, 3].

Post-stroke depression (PSD) is a common complication among stroke patients [4]. Approximately, one-third of stroke patients develop PSD [5, 6]. Common clinical manifestations of PSD include sadness, anxiety, emptiness, hopelessness or worthlessness, changes in eating and sleeping patterns, social withdrawal, reduced interest in previously enjoyable activities, irritability, difficulty concentrating, and even the expression of suicidal thoughts and plans [4]. PSD significantly reduces patients’ quality of life and recovery capacity, burdening families and society. Furthermore, PSD is associated with poor prognosis, an increased risk of recurrent stroke, and increased mortality among stroke patients [7]. With advancements in stroke treatment technology, the number of stroke survivors has also increased in recent years, leading to a greater incidence of PSD and exacerbated related adverse effects [8].

Although an increasing number of studies have focused on PSD, the diagnosis and treatment of this disease remain difficult. Therefore, we focused on the risk factors for PSD and avenues for its prevention. The risk factors for PSD are multifaceted and involve endogenous or exogenous factors. Endogenous factors are stroke-related, including the severity of the stroke, location of the lesion, physical disability, and cognitive impairment [9, 10]. Exogenous factors mainly involve social determinants, such as economic conditions and social support [11, 12].

According to social determinants of health theory, socioeconomic status (SES) is a key social determinant that affects a wide range of health outcomes through healthcare access, health literacy, and biological pathways [13]. A large body of literature has shown that low SES is associated with poor health outcomes. Freeman et al. reported a negative correlation between SES and depression [14]. Jiang et al. reported a relationship between SES and morbidity rates [15]. Studies on how SES relates to health outcomes have suggested that the association arises because those with high SES have better access to resources that promote health [16]. People with high SES also tend to better understand and follow health guidance and are likely to be exposed to risk factors that affect their health [15, 17].

Many studies have explored the relationship between SES and depression. However, studies on the relationship between SES and PSD are relatively rare, especially those focused on the Chinese population. As the world’s most populous developing country, China has the highest number of stroke-related cases and deaths [18]. According to the Global Burden of Disease Study 2019 results, China recorded 3.94 million new stroke cases in 2019 [19]. There is a considerable population of stroke survivors in China, and a significant number of these individuals are at risk of developing PSD. Therefore, this study used data from the China Health and Retirement Longitudinal Study (CHARLS) of the Chinese mainland population to explore the relationship between SES and PSD, aiming to identify the valuable factors influencing PSD and provide a theoretical foundation for creating a conducive environment for its prevention.

## Materials and methods

### Study design and participants

The CHARLS is a nationally representative longitudinal survey that enrolled a sample of middle-aged and older people from 450 villages and urban communities in 28 provinces in China. The CHARLS questionnaire covers various aspects of participants’ lives, including social, economic, physical, and mental health. The survey was first conducted from 2011 to 2012 as a large-scale baseline survey across the country, and subsequent follow-ups were conducted every 2–3 years. The CHARLS data are available on its official website (http://charls.pku.edu.cn/).

**Fig. 1:**
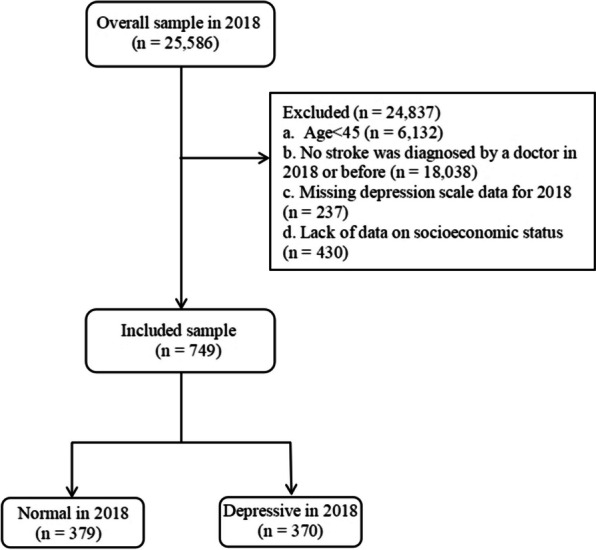
Flowchart of participants included in the analysis

This study used data from the fourth wave of the 2018 CHARLS database, which covers the period from June 2018 to March 2019 [20]. Participants aged 45 years and older were included in the analysis, while those who met any of the following criteria were excluded: (1) younger than 45, (2) had not been diagnosed with stroke in 2018 or prior, (3) lacked Center of Epidemiologic Studies Depression Scale-10 (CES-D-10) data in 2018, or (4) lacked data on SES indicators in 2018. We selected patients with stroke among the CHARLS participants by asking, “Have you been diagnosed with stroke by a doctor? (cerebral infarction and cerebral hemorrhage).” Participants who answered “yes” were defined as having had a stroke, while participants who answered “no” were defined as not having had a stroke. A total of 25,586 participants completed the CHARLS in 2018. We excluded 24,837 individuals from the overall sample per the above criteria. Ultimately, 749 individuals were included in this study. Additional details regarding the exclusion criteria are shown in Fig. 1.

### Measures

#### Depression assessment

Depression was assessed using CES-D-10. The 20-item version of the CES-D was initially developed by Andresen et al., who later revised it and created a simple 10-item scale [21]. Participants were asked 10 questions about their feelings or behavior over the past week. Each question had the same set of response options, including rarely or none of the time (< 1 day), some or a little of the time (1–2 days), occasionally or a moderate amount of the time (3–4 days), and most or all of the time (5–7 days). A four-point Likert scale was used to assign scores of 0, 1, 2, or 3 to the response options. For Question 5 (“I felt hopeful about the future”) and Question 8 (“I was happy”), the scoring was reversed, with “3, 2, 1, and 0” points assigned. The total score for the 10 items ranged from 0 to 30, with a score of 10 or higher indicating the presence of depression [22].

#### SES

The indicators used to assess SES often include education, occupation, housing tenure, income, and expenditure [15, 23]. Here, we focused on education attainment and household expenditure. We used the participants’ answers to categorize educational attainment into three levels: primary school or below, middle school, and high school or above. Household income is an important indicator of SES; however, because the questionnaire on household income in the CHARLS is very complex, we replaced it with a more easily measured indicator, household expenditure. Household expenditure is not independent of income and can better reflect income levels and economic conditions. In the CHARLS, household expenditure is measured by food expenditure, entertainment fees, utility bills, and communication and transportation costs. It is important to note that we excluded household food expenditure when exploring the relationship between expenditure and PSD. We divided the participants into five groups in each province by ranking them from lowest to highest in terms of annual household expenditure to avoid the influence of regional economic differences on SES. Ultimately, household expenditure was categorized into quintiles: poorest, poorer, middle, rich, and richest. Occupation type was not included in the study because it does not apply to people who are currently unemployed (such as stay-at-home mothers, jobless individuals, and retired people). Moreover, using occupational level as an SES indicator is not appropriate because the criteria for classifying occupational types are subjective and outdated [14].

#### Control variables

We selected the following variables as control variables according to the CHARLS survey and previous data:

Sociodemographic characteristics: (1) age (45–54, 55–64, 65–74, and 75 and older); (2) sex; (3) region (urban or rural); and (4) marital status (living with a spouse or partner or not living with a spouse or partner).

Health status: (1) had any chronic disease other than stroke; (2) self-reported health status; and (3) ability to perform activities of daily living (ADLs). Other chronic diseases included hypertension, dyslipidemia, diabetes, cancer, lung disease, liver disease, heart attack, kidney disease, stomach or other digestive diseases, memory-related diseases, arthritis or rheumatism, asthma, and emotional, nervous, or psychiatric problems. The assessment of ADL difficulties included dressing, bathing, eating, getting out of bed, going to the toilet, and controlling one’s bowel. The score ranged from “0” to “3,” with a score > 0 indicating mobility impairment [24].

Lifestyle measures included the following: (1) night-time sleep duration (≤ 6, 6–8, or ≥ 8 h); (2) smoking and drinking status; (3) life satisfaction (not satisfied, relatively satisfied, or very satisfied); and (4) physical and social activities. Participants were asked whether they consistently engaged in physical activity (either intense, moderate, or mild) for at least 10 min per week, and their responses were marked as “yes” or “no.” Additionally, participants were asked about their social activities over the past month.

### Statistical analyses

The included participants were divided into depressive and nondepressive groups, and statistical analyses were conducted using IBM SPSS Statistics v25. Descriptive statistics were used to analyze the participant characteristics. Categorical variables are presented as absolute numbers and proportions (%). The normality of continuous variables was tested using the Kolmogorov‒Smirnov method. Normally distributed data are presented as the mean ± standard deviation (x̄ ± s), while nonnormally distributed data are presented as the median or interquartile range. An analysis of variance or the Wilcoxon rank sum test was performed for continuous variables to compare the differences between the two groups, and the chi-square test was performed for categorical variables. Collinearity analysis was performed on all variables, and the results showed that the variance inflation factor values were all greater than 1 or close to 1. Multivariate logistic regression was used to analyze the specific influence of SES on depression symptoms in stroke patients. In addition to the crude models, multivariable-adjusted logistic regression analyses were conducted, adjusting for potential confounders such as sex, region, chronic diseases (other than stroke), self-reported health status, ADLs, night-time sleep duration, smoking, drinking, physical activity, social activity, and life satisfaction. Some data were missing from the control variable information, such as self-reported health status. We adopted the interpolation method to solve this problem. Stratified analysis by region type was performed to explore differences in the relationship between SES and PSD in different subgroups. Odds ratios (ORs) and 95% confidence intervals (CIs) were calculated, and bilateral *P* values < 0.05 were considered to indicate significance.

## Results

### Basic characteristics of the depressive and nondepressive groups

Table 1 presents the basic characteristics of the study participants. The participants comprised 383 (51.1%) men and 366 (48.9%) women, with a mean age of 65 ± 9 years. Most participants were within the age range of 65–74 years (39.0%), lived in rural areas (57.3%), lived with a spouse or partner (82.0%), had any chronic disease other than stroke (94.9%), reported poor self-reported health status (54.3%), and had no difficulties performing ADLs (60.9%). Additionally, most participants reported sleeping 6 h or less at night (60.1%), being nonsmokers (76.6%), abstaining from alcohol (74.2%), engaging in weekly physical activities (85.3%), not participating in social activities (55.9%), and having relatively satisfactory life satisfaction (55.1%). Among the 749 stroke patients included in the study, 370 (49.4%) experienced depressive symptoms.

**Table 1:** Comparison of sample characteristics between the depressive and nondepressive groups

	Total	Depression		χ^2^	* P* value
		No	Yes		
Sociodemographic characteristics					
**Sex, n (%)**				45.62	< 0.001***
Male	383 (51.1)	240 (62.7)	143 (37.3)		
Female	366 (48.9)	139 (38.0)	227 (62.0)		
**Age (in years), n (%)**				2.99	0.394
45–54	93 (12.4)	44 (47.3)	49 (52.7)		
55–64	247 (33.0)	133 (53.8)	114 (46.2)		
65–74	292 (39.0)	139 (47.6)	153 (52.4)		
≥ 75	117 (15.6)	63 (53.8)	54 (46.2)		
**Region, n (%)**				14.84	< 0.001***
Urban	320 (42.7)	188 (58.8)	132 (41.3)		
Rural	429 (57.3)	191 (44.5)	238 (55.5)		
**Marital status, n (%)**				3.13	0.077
No	135 (18.0)	59 (43.7)	76 (56.3)		
Yes	614 (82.0)	320 (52.1)	294 (47.9)		
**Health status**					
**Chronic diseases (other than stroke), n(%)**				5.09	0.024*
No	38 (5.1)	26 (68.4)	12 (31.6)		
Yes	711 (94.9)	353 (49.6)	358 (50.4)		
**Self-reported health status, n (%)**				66.98	< 0.001***
Good	73 (9.7)	58 (79.5)	15 (20.5)		
Fair	269 (35.9)	168 (62.5)	101 (37.5)		
Poor	407 (54.3)	153 (37.6)	254 (62.4)		
**ADL, n (%)**				47.99	< 0.001***
Normal	456 (60.9)	277 (60.7)	179 (39.3)		
Abnormal	293 (39.1)	102 (34.8)	191 (65.2)		
**Lifestyle**					
**Night-time sleep duration (in hours), n (%)**				27.34	< 0.001***
≤ 6	450 (60.1)	196 (43.6)	254 (56.4)		
6–8	125 (16.7)	86 (68.8)	39 (31.2)		
≥ 8	174 (23.2)	97 (55.7)	77 (44.3)		
**Smoking, n (%)**				10.15	0.001**
No	574 (76.6)	272 (47.4)	302 (52.6)		
Yes	175 (23.4)	107 (61.1)	68 (38.9)		
**Drinking, n (%)**				8.40	0.004**
No	556 (74.2)	264 (47.5)	292 (52.5)		
Yes	193 (25.8)	115 (59.6)	78 (40.4)		
**Physical activity, n (%)**				9.16	0.002**
No	110 (14.7)	41 (37.3)	69 (62.7)		
Yes	639 (85.3)	338 (52.9)	301 (47.1)		
**Social activity, n (%)**				2.63	0.105
No	419 (55.9)	201 (48.0)	218 (52.0)		
Yes	330 (44.1)	178 (53.9)	152 (46.1)		
**Life satisfaction, n (%)**				72.57	< 0.001***
Not satisfied	191 (30.1)	143 (74.9)	48 (25.1)		
Relatively satisfied	350 (55.1)	212 (60.6)	138 (39.4)		
Very satisfied	94 (14.8)	21 (22.3)	73 (77.7)		

*P < 0.05, **P < 0.01, and ***P < 0.001

Significant differences were observed between the depressive and nondepressive groups in terms of sex (χ^2^ = 45.62, *P* < 0.001), region (χ^2^ = 14.84, *P* < 0.001), chronic diseases (other than stroke) (χ^2^ = 5.09, *P* = 0.024), self-reported health status (χ^2^ = 66.98, *P* < 0.001), activities of daily living (χ^2^ = 47.99, *P* < 0.001), night-time sleep duration (χ^2^ = 27.34, *P* < 0.001), smoking (χ^2^ = 10.15, *P* = 0.001), drinking (χ^2^ = 8.40, *P* = 0.004), physical activity (χ^2^ = 9.16, *P* = 0.002), and life satisfaction (χ^2^ = 72.57, *P* < 0.001).

### Comparison of SES between the depressive and nondepressive groups

 Education attainment and household expenditure were used to assess the participants’ SES. As demonstrated in Table 2, 536 (71.6%) of the older adults had an education level of primary school or below, followed by middle school (18.3%) and high school or above (10.1%). In terms of SES, the incidence of depression was greater in middle-aged and older people with an education attainment of middle school. Participants with a household expenditure in the middle 20% were most likely to experience depression. Further analysis of SES showed that education attainment was significantly associated with the incidence of PSD (χ^2^ = 10.96, *P* = 0.004).

**Table 2:** Comparison of socioeconomic status between the depressive and nondepressive groups

	Total	Depression		χ^2^	* P* value
		No	Yes		
**Expenditure quintiles, n (%)**				3.86	0.426
1 (Poorest)	140 (18.7)	67 (47.9)	73 (52.1)		
2 (Poorer)	152 (20.3)	76 (50.0)	76 (50.0)		
3 (Middle)	146 (19.5)	68 (46.6)	78 (53.4)		
4 (Rich)	151 (20.2)	86 (57.0)	65 (43.0)		
5 (Richest)	160 (21.4)	82 (51.2)	78 (48.8)		
**Education, n (%)**				10.96	0.004**
Primary school or below	536 (71.6)	276 (51.5)	260 (48.5)		
Middle school	137 (18.3)	55 (40.1)	82 (59.9)		
High school or above	76 (10.1)	48 (63.2)	28 (36.8)		

**P < 0.01

### Logistic regression analysis of SES and PSD

Table 3 presents the results of the logistic regression analysis. Stroke patients with an education level of middle school had a greater risk of developing depression according to the unadjusted model (OR = 1.58, 95% CI: 1.08–2.32, *P* = 0.018). After adjusting for all control variables, the association between education attainment and depression in stroke patients remained significant (OR = 1.60, 95% CI: 1.03–2.51, *P* = 0.036; OR = 0.50, 95% CI: 0.28–0.88, *P* = 0.016). Thus, patients with a middle school education are 60% more likely to develop depression than those with a primary school education or below. However, the risk of developing depression decreased by 50% in stroke patients with a high school education or above compared with those with a primary school education or below.

**Table 3:** Logistic regression analysis of socioeconomic status and PSD

	Crude model		Adjusted model	
	OR	95% CI	OR	95% CI
Education (ref: primary school or below)				
Middle school	1.58*	1.08–2.32	1.60*	1.03–2.51
High school or above	0.62	0.38–1.02	0.50*	0.28–0.88
**Gender (ref: male)**			2.50***	1.69–3.68
**Region (ref: urban)**			1.74**	1.23–2.45
**Chronic diseases (other than stroke) (ref: no)**			1.21	0.52–2.83
**Self-reported health status (ref: good)**				
Fair			2.38*	1.19–4.75
Poor			4.22***	2.13–8.36
**ADL (ref: normal)**			2.02***	1.41–2.89
**Night-time sleep duration (in hours) (ref: ≤6)**				
6–8			0.43**	0.26–0.70
≥ 8			0.69	0.46–1.04
**Smoking (ref: no)**			0.90	0.57–1.427
**Drinking (ref: no)**			1.21	0.79–1.85
**Physical activity (ref: no)**			0.83	0.50–1.36
**Life satisfaction (ref: not satisfied)**				
Relatively satisfied			0.26***	0.15–0.45
Very satisfied			0.14***	0.08–0.25

*P < 0.05, **P < 0.01, and ***P < 0.001. *OR *Odds ratio, *95% CI *95% confidence interval. Crude model: without adjustment for any covariates. Adjusted model: adjusted for age, area, chronic diseases (other than stroke), self-reported health status, ADL, night-time sleep duration, smoking status, drinking status, physical activity, and life satisfaction

Sex, region, self-reported health status, ADLs, night-time sleep duration and life satisfaction were also found to influence the risk of depression in stroke patients. Women are approximately twice as likely to be depressed than men (OR = 2.50, 95% CI: 1.69–3.68, *P* < 0.001). Stroke patients living in rural areas were more likely to develop depression than those living in urban areas (OR = 1.74, 95% CI: 1.23–2.45, *P* = 0.002). Patients with poorer self-reported health status were more likely to develop depression (OR = 2.38, 95% CI: 1.19–4.75, *P* = 0.014; OR = 4.22, 95% CI: 2.13–8.36, *P* < 0.001). Participants with ADL difficulties were more likely to develop depression than those with a normal ability to perform ADLs (OR = 2.02, 95% CI: 1.41–2.89, *P* < 0.001). Patients who slept between 6 and 8 h per day had a lower risk of developing depression than those who slept fewer than 6 h (OR = 0.43, 95% CI: 0.26–0.72, *P* = 0.001). In terms of life satisfaction, stroke survivors who were relatively satisfied (OR = 0.26, 95% CI: 0.15–0.45, *P* < 0.001) or not satisfied (OR = 0.14, 95% CI: 0.08–0.25, *P* < 0.001) had a lower likelihood of developing depression than those who were very satisfied with their lives.

### Subgroup analysis of SES and PSD

We conducted a subgroup analysis of the relationship between SES and depression in stroke patients according to regional division. As shown in Table 4, after adjusting for all of the above variables, among rural participants, stroke patients with an education level of high school or above had lower rates of depression than those with an education level of primary school or below (OR = 0.44, 95% CI: 0.21–0.91, *P* = 0.027). However, there was no significant difference in depression risk between individuals with high and low household expenditures (*P* > 0.05). There were no significant differences in SES and PSD rates among the subgroup analysis variables in the urban sample.

**Table 4:** Logistic regression analysis of socioeconomic status and PSD among urban (*n* = 320) and rural (*n* = 429) residents in China, 2018

	Urban		Rural	
	OR (95% CI)	*P* value	OR (95% CI)	* P* value
**Education (ref: primary school or below)**				
Middle school	1.64 (0.83–3.25)	0.16	1.65 (0.89–3.08)	0.11
High school or above	0.63 (0.24–1.64)	0.34	0.44 (0.21–0.91)	0.027*
**Expenditure quintiles (ref: 1)**				
2 (Poorer)	0.74 (0.31–1.73)	0.48	0.90 (0.44–1.83)	0.77
3 (Middle)	1.20 (0.50–2.88)	0.69	1.13 (0.53–2.38)	0.76
4 (Rich)	0.46 (0.19–1.15)	0.10	0.92 (0.45–1.89)	0.83
5 (Richest)	0.47 (0.19–1.15)	0.10	1.01 (0.50–2.04)	0.99

*P < 0.05. Adjusted covariates: sex, self-reported health status, ADL, night-time sleep duration, smoking status, alcohol consumption, physical activity, and life satisfaction

## Discussion

Our large-scale systematic survey of Chinese individuals aged 45 years and older was conducted to analyze the association between SES and PSD, and provide guidance for stroke care policies. Our results demonstrate that the prevalence of depressive symptoms among middle-aged and older adults in China is 49.4%. As one of the main indicators of SES, education attainment is significantly correlated with the occurrence of PSD. Furthermore, the relationship between SES and PSD varied between urban and rural areas.

The incidence of depression is high among stroke patients. Research conducted by Stokman-Meiland et al. revealed that PSD is a prevalent complication after stroke, with 25–79% of stroke patients being diagnosed with PSD within 5 months after stroke [25]. However, the reported PSD incidence varies across studies. For instance, Jorgensen et al. reported that 25.4% of stroke patients developed depression within 2 years [26]. In a meta-analysis conducted by Ayerbe et al., the PSD incidence was reported to be 29%, with a cumulative incidence ranging from 39 to 52% within 5 years of stroke onset [27]. These variations can be attributed to differences in research methods and the complexity of diagnosing depression [28]. In our study, 49.4% of stroke patients experienced depressive symptoms; this proportion is significantly greater than the international average. Compared with stroke survivors in other countries, stroke survivors in China are more likely to experience the detrimental effects of PSD, highlighting the significance of studying the risk factors associated with PSD in this population.

In our study, education attainment had an independent influence on PSD. There is a strong link between education attainment and SES. Education is regarded as a bridge to acquire knowledge, improve skills, and broaden horizons, whereas SES concerns people’s position in society and their access to resources [29, 30]. Therefore, in this study, education attainment was taken as an observable indicator of SES. We found that the percentage of patients experiencing PSD decreased as the level of education increased. The proportion of patients who presented with PSD increased and then decreased. Many studies have concluded that individuals with higher education levels are less likely to develop PSD [31, 32]. This finding may be related to the following factors. First, educational attainment is a proxy for SES, and individuals with low SES may be more likely to experience brain defects and dysfunction due to a lack of financial support to meet their health needs. Second, education represents an individual’s ability to afford treatment for brain dysfunction after stroke, and patients with low education have fewer reserves to afford rehabilitation therapy interventions to restore or compensate the memory deficits and are more susceptible to depression [33]. Unlike previous studies, we found that the middle school-educated group was more likely to have PSD than the primary school-educated and below group, potentially because those with higher education levels are subject to more social pressures. Factors such as occupation and mindset may also affect this group [34, 35].

The mean annual income can also reflect SES. We divided the mean annual income into five classes for comparison; unfortunately, there were no significant differences between the groups. However, it has also been demonstrated that the mean annual income plays an important role in the development of PSD [36]. The greater the mean annual income is, the lower the likelihood of developing PSD. The fact that this study did not achieve the expected results may be related to participant memory bias; the data on income and consumption were derived from participant recall and might not be accurate. This finding is also related to the database we used.

Consumer spending was considered an independent prognostic factor influencing the occurrence of PSD in previous studies [37], which concluded that patients with higher levels of consumption had more interests derived from modes of regulation and were less prone to PSD. Partners of stroke patients also have a relatively light burden of care, which can provide positive emotional value and reduce the occurrence of depression [38, 39]. Our study categorized household expenditures into quintiles. The probability of depression was greater in quintiles 1, 2, and 3 than in quintiles 4 and 5. That is, people with low expenditure levels are more prone to depression than those with high expenditure levels. There was an overall decreasing trend in the occurrence of PSD, but the results were not significantly different. This finding may be related to differences in the consumption habits of people in different regions. Because of the large differences in consumption habits and consumption levels in different regions of China [40, 41], it is difficult to avoid this problem when selecting samples, and studies with larger sample sizes are needed.

In rural areas, there was a significant difference in the effect of education attainment on the occurrence of PSD, whereas there was no significant difference among urban residents. A prospective study of 155,722 individuals revealed that the low-education group had lower overall diet quality scores and higher rates of smoking and drinking, compared to the high-education group [42]. Lower levels of education were more frequently associated with adverse health behaviors [43]. Education influences various conditions from childhood, including exposure to community-level factors (such as living or working in healthier environments) and better access to health and social resources [44]. Therefore, educational attainment is strongly associated with an individual’s health status and disease prognosis. This association also explains why low educational attainment can be a risk factor for developing PSD. The difference in educational resources between rural and urban areas is large [45]. In rural areas, a high level of education is more likely to be supported by SES, especially in the 1970s, when several groups in rural areas had only an elementary school education level or lower (54.0%) [42]. Thus, we suggest that efforts should be made to increase social groups’ education level, tap and release more educational resources, resolutely implement compulsory education, actively popularize science in rural areas, pay attention to the health of groups, and establish the concept of health.

The effect of household expenditure on the occurrence of PSD is similar in both urban and rural areas; i.e., the greater the household expenditure, the lower the risk of PSD. PSD generally occurs in older adults. In a survey of 7,192 older adults (aged 65 and older) in households, the overall long-term care needs of older adults were found to show a rapidly increasing trend [46]. The ability to meet long-term care and other needs to improve disease prognosis is directly linked to the level of consumption. There are differences in this indicator between urban and rural areas, fundamentally due to significant differences in consumption levels, habits, and patterns among urban and rural residents [47, 48]. Zhang et al. also showed that older adults who live in rural areas are agricultural laborers, have low incomes, and are lonely and depressed and that they have a greater expected demand for community services [46]. Interestingly, in our study, the likelihood of PSD among the moderate consumption group was the highest among the respective groups, and both urban and rural areas agreed with this result, demonstrating the uniqueness of the moderate consumption group. However, there is currently insufficient research to explain this phenomenon, and the specific reasons need further exploration.

Our study identified several factors that can influence PSD incidence. Among the sex differences, female patients are more likely to experience PSD, possibly because female patients have more emotional changes and fluctuations than male patients. A short sleep duration can lead to negative emotions, reduced work energy, and increased anxiety and tension [49]. Patients with ADL limitations tend to rely on others for assistance, which can result in feelings of guilt and self-blame toward family and friends [50]. Those with poor self-reported health status [51, 52] often harbor negative attitudes toward their health, and some may experience prolonged suppressed emotions and disappointment related to their health in daily life.

This study has certain limitations. First, as an important indicator of SES, household income in the CHARLS database is not stable, whereas the level of household expenditure is more easily measured; therefore, the latter was used as an indicator to evaluate SES. As a result, our study lacked information on the effect of income-related indicators on PSD. Second, according to the characteristics of the study population, we selected two main indicators reflecting SES to investigate the relationship between SES and PSD, which may not fully represent the characteristics of SES. Third, the study relied on cross-sectional statistical data, limiting the ability to establish causal relationships between various risk factors and PSD occurrence. Further longitudinal research is necessary to confirm these relationships. Last, the study solely relied on the CES-D-10 for diagnosing depression and did not incorporate other diagnostic methods, potentially resulting in some undetected cases of depression.

## Conclusions

The results of this study revealed that SES is an important factor in PSD. Individuals with lower SES are more likely to have other physical and mental health problems, thus increasing the likelihood of PSD. This study also specifically examined the influence of SES on the incidence of PSD in urban and rural areas. Educational attainment, an indicator of SES, particularly influenced the prevalence of PSD in rural areas.

In addition, the results of this study revealed several factors that increase the likelihood of depression in stroke survivors, including poor self-reported health status, impaired self-care abilities, inadequate sleep, the presence of other chronic diseases, and low life satisfaction. Moreover, SES is not immediately changeable; thus, prevention strategies aimed at reducing the incidence of PSD can be developed through macroregulation, increased group education, and a reduction of known risk factors. Targeted interventions and support services can be developed to meet the specific needs of stroke survivors, especially those facing economic hardship, to reduce the risk and impact of PSD.

### Supplementary Information

**Supplementary Material 1.**

## Data Availability

The original datasets in the study can be found in the China Health and Retirement Longitudinal Study (CHARLS) (http://charls.pku.edu.cn/).
