# Supplementary material for: Association between socioeconomic status and post-stroke depression in middle-aged and older adults: results from the China health and retirement longitudinal study
Source: BMC Public Health. 2024 Apr 11;24:1007. doi: 10.1186/s12889-024-18503-z (PMC11010318; doi:10.1186/s12889-024-18503-z)
Supplement: Supplementary file 1 — Supplementary Material 1. [file 12889_2024_18503_MOESM1_ESM.docx]

Supplementary Material

Association Between Socioeconomic Status and Post-stroke Depression in Middle-aged and Older Adults: Results From the China Health and Retirement Longitudinal Study

Qianru Cai, Mengyi Qian, Meiling Chen*

*** Correspondence:** Meiling Chen: chenmeiling920@zcmu.edu.cn

**Supplementary Table 1.** Comparison of sample characteristics between the depressive and nondepressive groups among urban residents in China, 2018 (n=320).

|  | **Total** | **Depression** | | **χ^2^** | ***P* value** |
| --- | --- | --- | --- | --- | --- |
|  |  | **No** | **Yes** |  |  |
| **Sex, n (%)** |  | | | 14.74 | <0.001*** |
| Male | 167 (52.2) | 115 (68.9) | 52 (31.1) |  |  |
| Female | 153 (47.8) | 73 (47.7) | 80 (52.3) |  |  |
| **Age****, n (%)** |  | | | 1.50 | 0.68 |
| 45–54 | 33 (10.3) | 18 (54.5) | 15 (45.5) |  |  |
| 55–64 | 106 (33.1) | 67 (63.2) | 39 (36.8) |  |  |
| 65–74 | 121 (37.8) | 70 (57.9) | 51 (42.1) |  |  |
| ≥75 | 60 (18.8) | 33 (55.0) | 27 (45.0) |  |  |
| **Marital status, n (%)** |  | | | 3.71 | 0.054 |
| No | 57 (17.8) | 27 (47.4) | 30 (52.6) |  |  |
| Yes | 263 (82.2) | 161 (61.2) | 102 (38.8) |  |  |
| **Chronic diseases (other than stroke), n (%)** |  |  |  | 1.84 | 0.18 |
| No | 16 (5.0) | 12 (75.0) | 4 (25.0) |  |  |
| Yes | 304 (95.0) | 176 (57.9) | 128 (42.1) |  |  |
| **Self-reported health status, n (%)** |  |  |  | 29.00 | <0.001*** |
| Good | 36 (11.3) | 31 (86.1) | 5 (13.9) |  |  |
| Fair | 136 (42.5) | 92 (67.6) | 44 (32.4) |  |  |
| Poor | 148 (46.3) | 65 (43.9) | 83 (56.1) |  |  |
| **ADL, n (%)** |  |  |  | 20.46 | <0.001*** |
| Normal | 204 (63.7) | 138 (68.1) | 65 (31.9) |  |  |
| Abnormal | 116 (36.3) | 49 (42.2) | 67 (57.8) |  |  |
| **Night sleep duration (in hours), n (%)** |  |  |  | 20.63 | <0.001*** |
| ≤6 | 203 (63.4) | 100 (49.3) | 103 (50.7) |  |  |
| 6–8 | 61 (19.1) | 46 (75.4) | 15 (24.6) |  |  |
| ≥8 | 56 (17.5) | 42 (75.0) | 14 (25.0) |  |  |
| **Smoking, n (%)** |  |  |  | 3.71 | 0.054 |
| No | 247 (77.2) | 138 (55.9) | 109 (44.1) |  |  |
| Yes | 73 (22.8) | 50 (68.5) | 23 (31.5) |  |  |
| **Drinking, n (%)** |  |  |  | 4.31 | 0.038* |
| No | 222 (69.4) | 122 (55.0) | 100 (45.0) |  |  |
| Yes | 98 (30.6) | 66 (67.3) | 32 (32.7) |  |  |
| **Physical activity, n (%)** |  |  |  | 8.52 | 0.004** |
| No | 40 (12.5) | 15 (37.5) | 25 (62.5) |  |  |
| Yes | 280 (87.5) | 173 (61.8) | 107 (38.2) |  |  |
| **Social activity, n (%)** |  |  |  | 0.52 | 0.47 |
| No | 162 (50.6) | 92 (56.8) | 70 (43.2) |  |  |
| Yes | 158 (49.4) | 96 (60.8) | 62 (39.2) |  |  |
| **Life satisfaction, n (%)** |  |  |  | 54.06 | <0.001*** |
| Not satisfied | 41 (12.8) | 4 (9.8) | 37 (90.2) |  |  |
| Relatively satisfied | 183 (57.2) | 110 (60.1) | 73 (39.9) |  |  |
| Very satisfied | 86 (30.0) | 74 (77.1) | 22 (22.9) |  |  |
| **Expenditure Quintiles, n (%)** |  |  |  | 5.61 | 0.23 |
| 1 (Poorest) | 55 (17.2) | 28 (50.9) | 27 (49.1) |  |  |
| 2 (Poorer) | 68 (21.3) | 41 (60.3) | 27 (39.7) |  |  |
| 3 (Middle) | 60 (18.8) | 30 (50.0) | 30 (50.0) |  |  |
| 4 (Rich) | 65 (20.3) | 43 (66.2) | 22 (33.8) |  |  |
| 5 (Richest) | 72 (22.5) | 46 (63.9) | 26 (36.1) |  |  |
| **Education, n (%)** |  |  |  | 4.24 | 0.12 |
| Primary school or below | 224 (70.0) | 137 (61.2) | 87 (38.8) |  |  |
| Middle school | 65 (20.3) | 31 (47.7) | 34 (52.3) |  |  |
| High school or above | 31 (9.7) | 20 (64.5) | 11 (35.5) |  |  |

**P* < 0.05, ***P* < 0.01, and ****P* < 0.001.

**Supplementary Table 2.** Comparison of sample characteristics between the depressive and nondepressive groups among rural residents in China, 2018 (n=429).

|  | **Total** | **Depression** | | **χ^2^/t** | ***P* value** |
| --- | --- | --- | --- | --- | --- |
|  |  | **No** | **Yes** |  |  |
| **Sex, n (%)** |  | | | 31.38 | <0.001*** |
| Male | 216 (50.3) | 125 (57.9) | 91 (42.1) |  |  |
| Female | 213 (49.7) | 66 (31.0) | 147 (69.0) |  |  |
| **Age, n (%)** |  | | | 3.06 | 0.38 |
| 45–54 | 60 (14.0) | 26 (43.3) | 34 (56.7) |  |  |
| 55–64 | 141 (32.9) | 66 (46.8) | 75 (53.2) |  |  |
| 65–74 | 171 (39.9) | 69 (46.8) | 75 (53.2) |  |  |
| ≥75 | 57 (13.3) | 30 (52.6) | 27 (47.4) |  |  |
| **Marital status, n (%)** |  | | | 0.47 | 0.49 |
| No | 78 (18.2) | 32 (41.0) | 46 (59.0) |  |  |
| Yes | 351 (81.8) | 159 (45.3) | 192 (54.7) |  |  |
| **Chronic diseases (other than stroke), n (%)** |  |  |  | 3.43 | 0.064 |
| No | 22 (5.1) | 14 (63.6) | 8 (36.4) |  |  |
| Yes | 407 (94.9) | 177 (43.5) | 230 (56.5) |  |  |
| **Self-reported health status, n (%)** |  |  |  | 31.93 | <0.001*** |
| Good | 36 (8.4) | 26 (72.2) | 10 (27.8) |  |  |
| Fair | 134 (31.2) | 77 (57.5) | 57 (42.5) |  |  |
| Poor | 259 (60.4) | 88 (34.0) | 171 (66.0) |  |  |
| **ADL, n (%)** |  |  |  | 25.93 | <0.001*** |
| Normal | 252 (58.7) | 138 (54.8) | 114 (45.2) |  |  |
| Abnormal | 177 (41.3) | 53 (29.9) | 124 (70.1) |  |  |
| **Night sleep duration (in hours),**  **n (%)** |  |  |  | 11.78 | 0.003** |
| ≤6 | 247 (57.6) | 96 (38.9) | 151 (57.6) |  |  |
| 6–8 | 64 (14.9) | 40 (62.5) | 24 (37.5) |  |  |
| ≥8 | 118 (27.5) | 55 (46.6) | 63 (53.4) |  |  |
| **Smoking, n (%)** |  |  |  | 6.99 | 0.008** |
| No | 327 (76.2) | 134 (41.0) | 193 (59.0) |  |  |
| Yes | 102 (23.8) | 57 (55.9) | 45 (44.1) |  |  |
| **Drinking, n (%)** |  |  |  | 2.46 | 0.117 |
| No | 334 (77.9) | 142 (42.5) | 192 (57.5) |  |  |
| Yes | 95 (22.1) | 49 (51.6) | 46 (48.4) |  |  |
| **Physical activity, n (%)** |  |  |  | 1.84 | 0.174 |
| No | 70 (16.3) | 26 (37.1) | 44 (62.9) |  |  |
| Yes | 359 (83.7) | 165 (46.0) | 194 (54.0) |  |  |
| **Social activity, n (%)** |  |  |  | 1.16 | 0.282 |
| No | 257 (59.9) | 109 (42.4) | 148 (57.6) |  |  |
| Yes | 172 (40.1) | 82 (47.7) | 90 (52.3) |  |  |
| **Life satisfaction, n (%)** |  |  |  | 30.86 | <0.001*** |
| Not satisfied | 82 (19.1) | 17 (20.7) | 65 (79.3) |  |  |
| Relatively satisfied | 220 (51.3) | 98 (44.5) | 122 (55.5) |  |  |
| Very satisfied | 127 (29.6) | 76 (59.8) | 51 (40.2) |  |  |
| **Expenditure Quintiles, n (%)** |  |  |  | 1.26 | 0.87 |
| 1 (Poorest) | 76 (17.7) | 35 (46.1) | 41 (53.9) |  |  |
| 2 (Poorer) | 88 (20.5) | 39 (44.3) | 49 (55.7) |  |  |
| 3 (Middle) | 82 (19.1) | 33 (40.2) | 49 (59.8) |  |  |
| 4 (Rich) | 91 (21.2) | 44 (48.4) | 47 (51.6) |  |  |
| 5 (Richest) | 92 (21.4) | 40 (43.5) | 52 (56.5) |  |  |
| **Education, n (%)** |  |  |  | 9.36 | 0.009** |
| Primary school or below | 312 (72.7) | 139 (44.6) | 173 (55.4) |  |  |
| Middle school | 72 (16.8) | 24 (33.3) | 48 (66.7) |  |  |
| High school or above | 45 (10.5) | 28 (62.2) | 17 (37.8) |  |  |

***P* < 0.01, ****P* < 0.001.
